# Supplementary figures and images for: Genetic profiling of synchronous pituitary corticotroph adenomas
Source: Pituitary. 2025 Jun 22;28(4):77. doi: 10.1007/s11102-025-01549-6 (PMC12183135; doi:10.1007/s11102-025-01549-6)

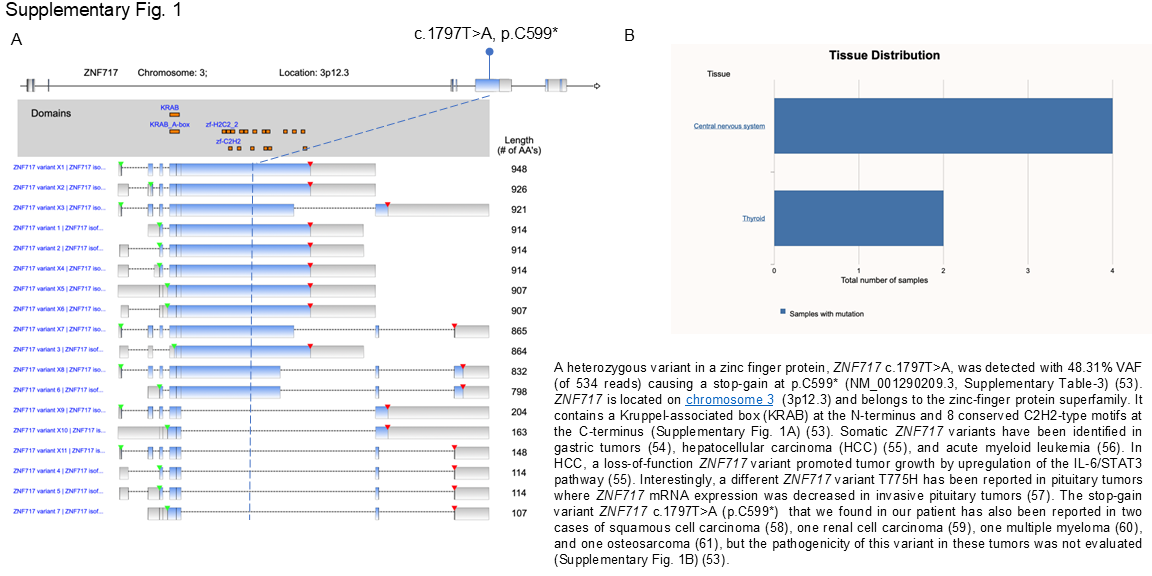

Supplement: Supplementary file 4 — Supplementary Material 4 [file 11102_2025_1549_MOESM4_ESM.tif]

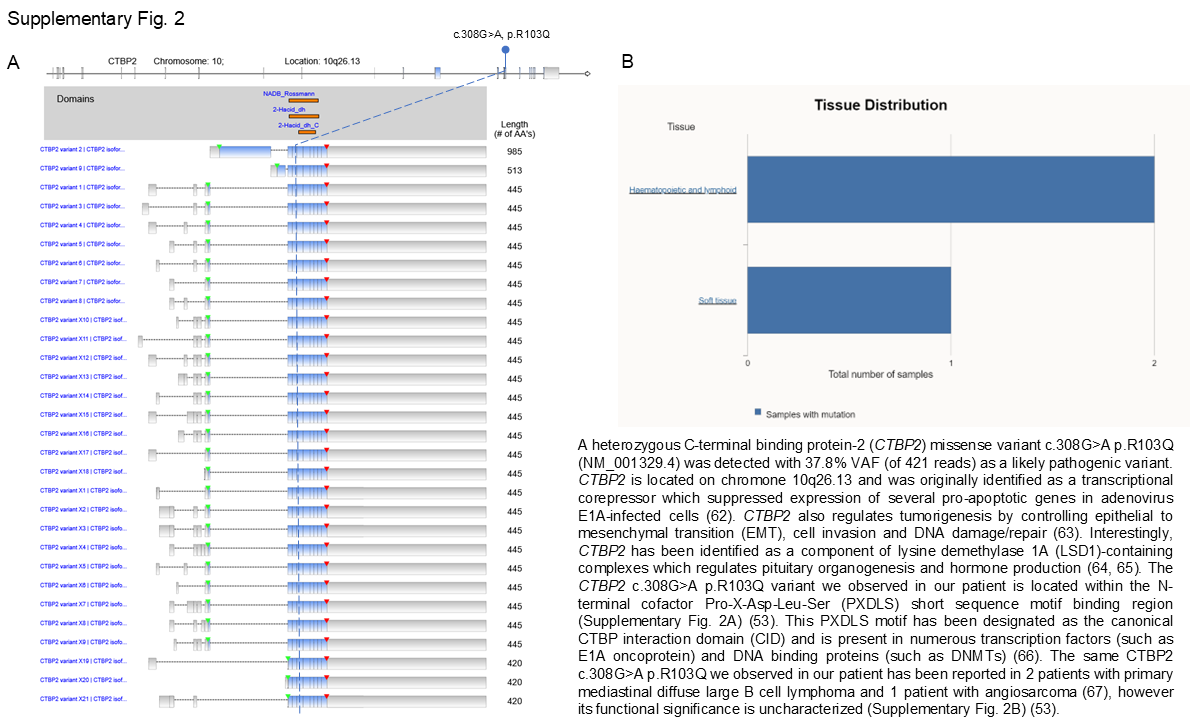

Supplement: Supplementary file 5 — Supplementary Material 5 [file 11102_2025_1549_MOESM5_ESM.tif]

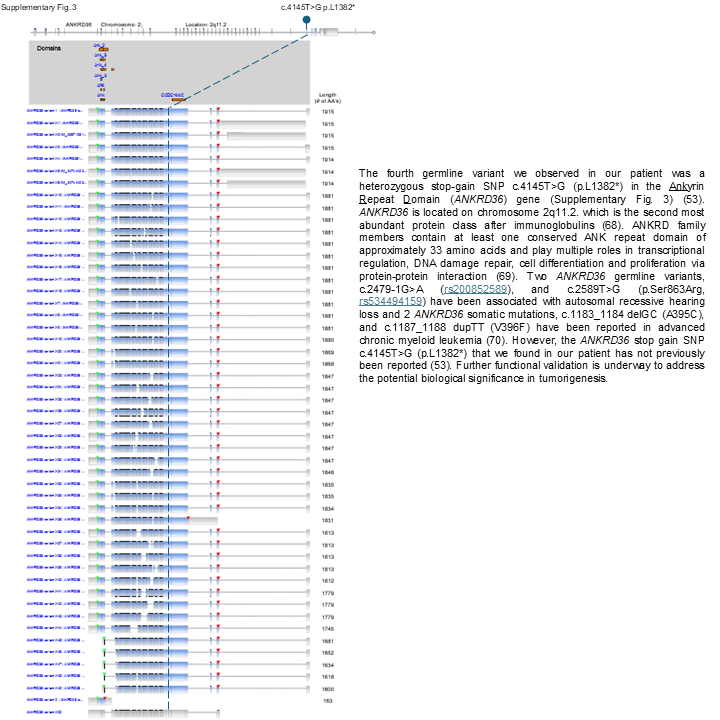

Supplement: Supplementary file 6 — Supplementary Material 6 [file 11102_2025_1549_MOESM6_ESM.tif]
